# Utility of absolute apparent diffusion coefficient and chemical‐shift imaging versus CT attenuation for predicting malignancy from percutaneous bone biopsies

**DOI:** 10.1002/jmrs.463

**Published:** 2021-02-19

**Authors:** Ronnie Sebro, S. Sharon Ashok

**Affiliations:** ^1^ Department of Radiology University of Pennsylvania Philadelphia Pennsylvania USA; ^2^ Department of Orthopaedic Surgery University of Pennsylvania Philadelphia Pennsylvania USA; ^3^ Department of Biostatistics, Epidemiology and Informatics University of Pennsylvania Philadelphia Pennsylvania USA; ^4^ Department of Genetics University of Pennsylvania Philadelphia Pennsylvania USA

**Keywords:** Bone biopsy, chemical‐shift imaging, CT attenuation, diffusion‐weighted imaging, MRI

## Abstract

**Introduction:**

Bone lesions are sometimes detected on computed tomography studies, and biopsies are performed to evaluate whether these are malignant. The aim of the study is to evaluate whether chemical‐shift imaging (CSI) and diffusion‐weighted imaging (DWI) magnetic resonance imaging (MRI) are more informative than the CT attenuation for predicting malignancy.

**Methods:**

Retrospective analysis of 86 patients who underwent both diagnostic CT, CSI MRI and DWI MRI within 6 weeks prior to bone biopsy at a tertiary care academic institution between 01/01/2010 and 03/01/2020. The CT attenuation, signal intensity on in‐phase sequences (SIIP), signal intensity on out‐of‐phase sequences (SIOP), signal intensity ratio (SIR = SIOP/SIIP) and the apparent diffusion coefficient (ADC) of the lesions over the region of the biopsy tract were measured.

**Results:**

A threshold CT attenuation of 157 Hounsfield Units (HU) had a sensitivity of 47.7%, specificity of 83.3% and area under the curve (AUC) of 0.59. A threshold ADC of 793 × 10^−6^ mm^2^/s had a sensitivity of 75.8%, specificity of 85.7% and AUC of 0.83 to predict whether a bone biopsy would detect malignancy. A threshold SIR of 0.949 had a sensitivity of 77.8%, specificity of 77.8% and AUC of 0.81 to predict whether a bone biopsy would detect malignancy. ADC (*P* = 0.029) and SIR (*P* = 0.009) were significantly better than CT attenuation. There was no predictive difference between SIR and ADC (*P* = 0.742).

**Conclusions:**

The CT attenuation of a lesion is a poor predictor of malignancy in bone lesions. CSI and DWI are significantly better for predicting malignancy.

## Graphical abstract

Diffusion‐weighted imaging (DWI) and Chemical‐shift imaging (CSI) from MRI are superior to CT attenuation for determining whether a bone lesion is malignant or benign. MRI may play a role in the evaluation of whether a bone lesion should be biopsied. Although CT attenuation may differ between benign and malignant sclerotic bone lesions, CT attenuation has limited ability to predict whether a bone lesion that is biopsied (suspicious for malignancy) is benign or malignant.

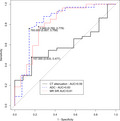

## Introduction

Computed tomography (CT), magnetic resonance imaging (MRI) and other imaging modalities are often used for staging and surveillance in patients with suspected primary or metastatic bone malignancies.^1^, ^2^, ^3^, ^4^, ^5^ While advances in imaging techniques have allowed for the detection of bone lesions on staging studies, bone biopsies are still required for confirmation of malignancy, and to provide tissue for additional histopathological studies.^6^, ^7^, ^8^, ^9^, ^10^, ^11^ Systemic treatment changes the imaging appearance of malignant bone lesions on CT^12^ and MRI.^13^ After systemic treatment, some osteoblastic lesions show increased CT attenuation and others show decreased CT attenuation.^12^ Systemic treatment results in increased signal drop‐out in out‐of‐phase chemical‐shift imaging MRI (CSI‐MRI) sequences.^13^ CT studies used for staging/surveillance also cannot differentiate a treated lesion from a viable untreated lesion.^14^ Because treatment history may often be unknown, there is a need for an imaging test that can be used to help predict whether a diagnostic bone biopsy will be show malignancy.

Absolute apparent diffusion coefficient (ADC) values from diffusion‐weighted imaging MRI (DWI‐MRI) studies are often used as an imaging biomarker for increased cellularity in soft tissue tumours,^15^, ^16^, ^17^ and have been used to assess response to chemotherapy. However, this has been less well studied for the evaluation of bone tumours. CSI‐MRI can be used to detect replacement of normal fatty marrow by tumour.^13^ The main hypothesis of this manuscript is that in patients with diffuse osseous malignancy, the ADC values from DWI‐MRI, and signal intensity ratios (SIR) from CSI‐MRI could be used to identify the optimal lesion for biopsy. The aim of this study is to evaluate whether ADC values and SIRs predict malignancy in diagnostic bone biopsies better than computed tomography (CT) attenuation values.

## Materials and Methods

The study protocol was reviewed and approved by the University of Pennsylvania Institutional Review Board (IRB) and the need for signed informed consent from each patient was waived.

### Inclusion and exclusion criteria

All consecutive bone biopsies on patients treated at a tertiary care academic healthcare institution (comprised of 3 hospitals) between 01/01/2010 and 03/01/2020 were reviewed. Patients were included if they had both (a) a magnetic resonance imaging (MRI) study with diffusion‐weighted imaging and chemical‐shift imaging and (b) a diagnostic CT of the biopsied lesion within 6 weeks prior to the bone biopsy. A 6‐week time period was chosen because clinicians (both community and academic) were generally able to read the imaging report showing the lesion, and have the biopsy scheduled and performed on the patient within 6 weeks after the imaging study was performed. Patients were excluded if the diffusion‐weighted sequence or the chemical‐shift sequences did not completely cover the lesion of interest, or if the bone biopsy was terminated before obtaining a bone biopsy sample. Patient age at the time of the biopsy, sex, type of cancer, lesion location and lesion size were recorded.

### Bone biopsies

Bone biopsies were performed by an American Board of Radiology (ABR) certified radiologist. Of the biopsies, 57 (66.3%) were CT‐guided and 29 (33.7%) were fluoroscopically guided. Biopsies were performed using either a 12G coaxial/13G or 14G coaxial/15G Bonopty biopsy system (AprioMed, Uppsala, Sweden) biopsy set; or a 11G, 11G coaxial/13 G Arrow^®^ OnControl^®^ Powered Bone Access System (Teleflex, Texas). A variable number (1–9) of core biopsies were obtained. The biopsy technique was reviewed by a musculoskeletal radiologist and confirmed to traverse the lesion. Biopsies were reviewed by a pathologist and classified as benign or malignant (primary or metastatic). The patients were followed for at least 9 months to evaluate whether these biopsies were false negative biopsies (based on lesion growth/change with therapy).

### CT attenuation

**Figure 1:**
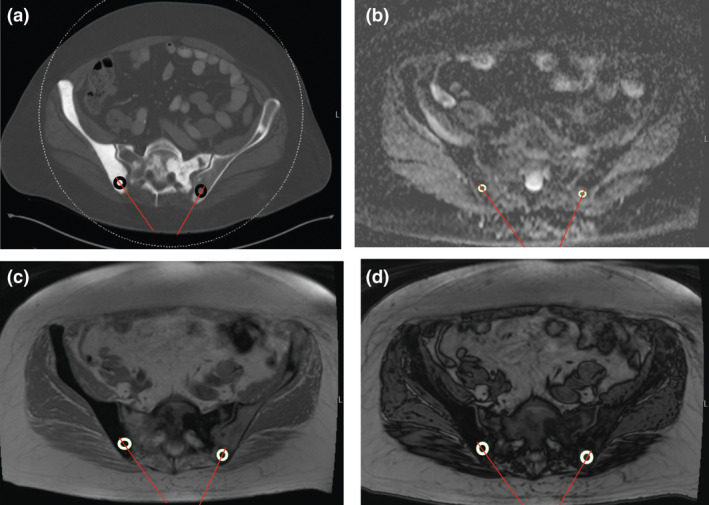
(A) Measurement of mean CT attenuation of lesions in the right and left posterior ilium in a 75‐year‐old man with suspected castrate‐resistant metastatic prostate cancer. The lesion in the right ilium had a CT attenuation of 1063 HU. The lesion in the left posterior ilium had a CT attenuation of 387 HU. Red lines show the biopsy needle paths. (B) Measurement of the signal intensity of lesions in the right and left posterior ilium on ADC MRI sequence in a 75‐year‐old man with suspected castrate‐resistant metastatic prostate cancer. The lesion in the right ilium had a mean ADC of 175 mm^2^/s and the lesion in the left posterior ilium had a mean ADC of 921 mm^2^/s. Red lines show the biopsy needle paths. (C) Measurement of the signal intensity of lesions in the right and left posterior ilium on in‐phase MRI in a 75‐year‐old man with suspected castrate‐resistant metastatic prostate cancer. The lesion in the right ilium had a mean signal intensity of 9.86 and the lesion in the left posterior ilium had a mean signal intensity of 41.46. Red lines show the biopsy needle paths. (D) Measurement of the signal intensity of lesions in the right and left posterior ilium on out‐of‐phase MRI sequence in a 75‐year‐old man with suspected castrate‐resistant metastatic prostate cancer. The lesion in the right ilium had a mean signal intensity of 6.42 (SIR 0.65) and the lesion in the left posterior ilium had a mean signal intensity of 50.59 (SIR 1.22). Red lines show the biopsy needle paths

The CT attenuation was measured on a diagnostic CT scan performed within 6 weeks prior to the biopsy. CT‐guided biopsies performed using a Siemens SOMATOM Definition AS 128 slice CT scanner (Siemens Healthineers, Malvern, PA) using 100–120 kVp and variable tube current. A circular region of interest (ROI) was placed over the region of the lesion that was biopsied, centred around the biopsy tract, taking care to avoid the bony cortex. For fluoroscopically guided biopsies, a musculoskeletal radiologist used anatomic landmarks to identify the expected biopsy tract on CT, and the measurements were taken over the region of the biopsy tract. The mean CT attenuation in Hounsfield units (HU) of the lesion was recorded (Fig. 1A).

### MRI measurements

Each patient had to have a MRI with diffusion‐weighted imaging and chemical‐shift imaging within 6 weeks prior to the bone biopsy, and the biopsied bone lesion had to be completely visualised on the ADC map and on chemical‐shift imaging sequences. MRIs were performed using 1.5T General Electric (GE) Signa (General Electric, Milwaukee, WI), 1.5T (Siemens Avanto) or 3.0T (Siemens Skyra) Siemens (Siemens Healthineers, Erlangen Germany) MRI scanners. Studies were obtained with the patient in the supine position. A body coil was used. The diffusion‐weighted images were obtained at *b* = 0, 400 and 800 s/mm^2^ gradients. The matrix was 128 × 128; NEX 1.0–2.0; field of view (FOV) 20 cm; slice thickness 4–6 mm; slice gap 0; diffusion direction all directions; repetition time (TR) 2650–6900 ms; and echo time (TE) 56–82 ms. The ADC map was obtained from DWI‐MRI. A ROI was placed over the region of the lesion that was biopsied, centred around the biopsy tract and the ADC in mm^2^/s was recorded (Fig. 1B).

The signal intensity ratios (SIRs) were measured from the CSI‐MRI sequences. The chemical‐shift gradient sequences were TR (6.24–184 ms), and TE (4.17–4.76 ms) for the in‐phase sequences; and TR (6.24–184 ms) and TE (2.08–2.38 ms) for the out‐of‐phase sequences. A ROI was placed over the region of the lesion that was biopsied, centred around the biopsy tract, and the signal intensity on the in‐phase sequences (SIIP) was recorded (Fig. 1C). The signal intensity on the out‐of‐phase sequences (SIOP) was calculated similarly by measuring the signal intensity in the region of the lesion that was biopsied, centred around the biopsy tract, using the out‐of‐phase sequences (Fig. 1D). The ratio of the SIOP to the SIIP was the SIR (Fig. 1D).

### Statistical analyses

The inter‐rater and intra‐rater reliability of the CT and MRI measurements were assessed using the intra‐class correlation coefficient (ICC) calculated on a random subset of 15 patients/lesions. The inter‐rater and intra‐rater reliability of the CT and MRI measurements were categorised following the guidelines proposed by Cicchetti.^18^

Both MRI and CT measurements were obtained by a musculoskeletal radiologist with 7 years of experience. A trained research assistant obtained measurements for the evaluation of inter‐rater reliability. Quantitative clinical and demographic variables were compared using t‐tests with unequal variances. Qualitative clinical and demographic characteristics were compared using Fisher’s exact test. Correlations between ADC values, SIRs and CT attenuation values were evaluated using Pearson’s correlation coefficients. Receiver operator characteristic (ROC) analyses were used to evaluate CT attenuation, SIRs and ADC values as predictors of malignancy. Accuracy, sensitivity, specificity and area under the curve (AUC) were calculated and reported. ROC curves were compared using DeLongs’ test. The analysis was repeated, stratified by whether the patient received systemic chemotherapy within 6 weeks prior to the biopsy. Statistical analyses were performed using Rv3.4 (Vienna, Austria). Statistical tests were two‐sided and *P*‐values < 0.05 were considered statistically significant.

## Results

The data from consecutive bone biopsies performed between 01/01/2010 and 03/01/2020 at a tertiary academic centre (comprised of 3 hospitals) was reviewed. The final study data comprised of 86 patients/biopsies who satisfied inclusion and exclusion criteria, 47.7% male and 52.3% female. The median age (range) of patients undergoing biopsies in the study was 66 (21–93) years (Table 1). Biopsies were most often performed for the detection of metastases rather than primary bone tumours, with prostate (*n* = 7, 8.1%), breast (*n* = 14, 16.3%) and lymphoma (11, 12.8%), being the most common cancers. 55 (64.0%) biopsies were positive for malignancy, and the other 31 (36.0%) were negative for malignancy. Clinical follow‐up of all biopsies, showed that there were no false negative biopsies. One patient underwent a repeat biopsy of the same lesion and had the same histopathological result that was negative for malignancy.

**Table 1:** Demographic and clinical characteristics of patients undergoing bone biopsies

Variable	All (*N* = 86)	Positive biopsies (*N* = 55)	Negative biopsies (*N* = 31)	*P*‐value
Age in years (SD)	62.5 (14.0)	63.71 (14.9)	60.4 (12.3)	0.264
Male sex, *n* (%)	41 (47.7%)	26 (47.3%)	15 (48.4%)	1.00
Diagnoses (*n*, %)				
Bladder	4 (4.7%)	4 (7.3%)	0 (0.0%)	0.048
Breast	14 (16.3%)	13 (23.6%)	1 (3.2%)	
Lung	3 (3.5%)	2 (3.6%)	1 (3.2%)	
Prostate	7 (8.1%)	3 (5.5%)	4 (12.9%)	
Lymphoma	11 (12.8%)	6 (10.9%)	5 (16.1%)	
Other	47 (54.7%)	27 (49.1%)	20 (64.5%)	
Lesion location (*n*, %)				
Thoracic spine	8 (9.3%)	7 (12.7%)	1 (3.2%)	0.406
Lumbar spine	7 (8.1%)	5 (9.1%)	2 (6.5%)	
Pelvis	56 (65.1%)	33 (60.0%)	23 (74.2%)	
Sacrum	3 (3.5%)	3 (5.5%)	0 (0.0%)	
Other	12 (14.0%)	7 (12.7%)	5 (16.1%)	
Lesion size in cm (SD)	1.8 (1.3)	1.9 (1.4)	1.6 (0.6)	0.270
Number of cores (SD)	2.11 (1.6)	2.32 (1.7)	1.77 (1.4)	0.115
Gauge biopsy				
11	21 (24.4%)	12 (21.8%)	9 (29.0%)	0.150
13	26 (30.2%)	19 (34.5%)	7 (22.6%)	
15	13 (15.1%)	11 (20.0%)	2 (6.5%)	
Unknown	26 (30.2%)	13 (23.6%)	13 (41.9%)	
CT attenuation in	312.9	303.5	347.5	0.641
Hounsfield units (HU) (SD)	(271.9)	(270.1)	(287.9)	
ADC in mm^2^/s (SD)	912.2 (545.3)	1076 (476.4)	526.6 (515.3)	0.002
SIR (SD)	0.913 (0.42)	1.08 (0.38)	0.65 (0.35)	5.39 × 10^−4^

ADC, attenuation diffusion coefficient; CT, computed tomography; HU, Hounsfield Units; SIR, signal intensity ratio.

There was excellent intra‐ and inter‐rater reliability for the CT and MRI measurements.^18^ The intra‐rater reliability for the CT attenuation, MRI ADC, MRI SIIP and MRI SIOP were ICC = 0.89 (*P* = 5.0 × 10^−8^), ICC = 0.97 (*P* = 5.3 × 10^−14^), ICC = 0.95 (*P* = 1.7 × 10^−11^) and ICC = 0.96 (*P* = 4.5 × 10^−12^), respectively. The inter‐rater reliability for the CT attenuation, MRI ADC, MRI SIIP and MRI SIOP were all greater than 0.9 (*P* < 0.001).

The lesion size was not statistically significantly different in patients with biopsies positive for malignancy compared to patients with negative biopsies (*P* = 0.270). Needle gauge and number of passes were also not significantly different in patients with biopsies positive for malignancy compared to patients with negative biopsies (Table 1). The proportion of biopsies positive for malignancy was not significantly different for lesions biopsied using fluoroscopic‐guidance compared with lesions biopsied using CT‐guidance (*P* = 0.080). There was no significant correlation between CT attenuation and ADC (*r *= −0.12, *P* = 0.458); no significant correlation between CT attenuation and the SIR (*r *= −0.05, *P* = 0.787) and no significant correlation between the SIR and ADC (*r* = 0.08, *P* = 0.626).

**Figure 2:**
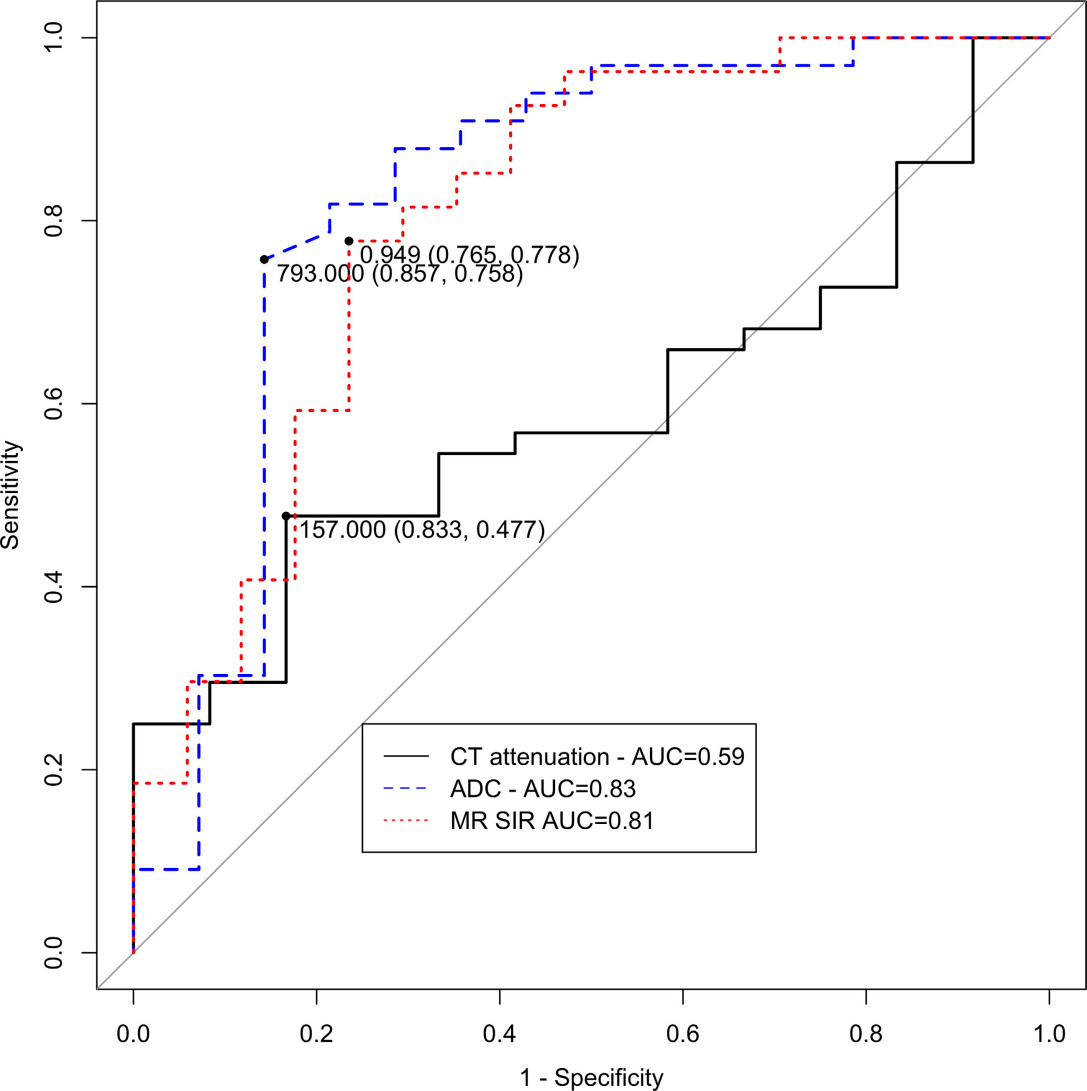
Receive Operator Characteristic (ROC) curves comparing the diagnostic performance of magnetic resonance (MR) chemical shift signal intensity ratio (SIR), MR apparent diffusion coefficient (ADC) and computed tomography (CT) attenuation for predicting malignancy from bone biopsies. AUC = area under the curve

A threshold CT attenuation of 157 Hounsfield Units (HU) had a sensitivity of 47.6%, specificity of 83.3% and AUC of 0.59 for predicting malignancy from bone biopsies. A threshold ADC of 793 × 10^−6 ^mm^2^/s from DWI‐MRI had a sensitivity of 75.8%, specificity of 85.7% and AUC of 0.83 for predicting malignancy from bone biopsies (Fig. 2). A threshold SIR of 0.949 from CSI‐MRI had a sensitivity of 77.8%, specificity of 77.8% and AUC of 0.81 for predicting malignancy from bone biopsies. ADC (*P* = 0.029) and SIR (*P* = 0.009) were significantly better than CT attenuation for predicting malignancy from bone biopsies. We found no significant predictive difference between the SIR and ADC (*P* = 0.513).

The analysis was repeated – stratified by whether the patient had ever received prior systemic treatment for cancer, or whether the patient had never received any treatment for cancer. For patients that had previous systemic chemotherapy, we found that the CT attenuation was not as predictive as the ADC or SIR in this analysis. A threshold ADC of 555 × 10^−6^ mm^2^/s and a threshold SIR of 0.792 had sensitivity of 100.0% for predicting malignancy from bone biopsies (Table 2). However, there was no significant predictive difference between the CT attenuation and ADC (*P* = 0.444) or SIR (*P* = 0.096). There was no significant predictive difference between ADC and SIR (*P* = 0.742).

**Table 2:** Diagnostic performance of CT attenuation, ADC and chemical‐shift MRI for predicting positive bone biopsies

All	Threshold	Sensitivity	Specificity	AUC
CT attenuation	157 HU	47.7	83.3	0.59
ADC	793.0 × 10^−6^ mm^2^/s	75.8	85.7	0.83
SIR	0.949	77.8	77.8	0.81
Untreated lesions				
CT attenuation	157 HU	54.5	100.0	0.66
ADC	793.0 × 10^−6^ mm^2^/s	75.0	87.5	0.83
SIR	0.949	76.2	70.0	0.73
Prior treatment				
CT attenuation	109.7 HU	90.9	50.0	0.57
ADC	555.0 × 10^−6^ mm^2^/s	100.0	83.3	0.85
SIR	0.792	100.0	71.4	0.91

ADC, attenuation diffusion coefficient; CT, computed tomography; HU, Hounsfield Units; SIR, signal intensity ratio.

In patients that never received any systemic treatment, the SIR performed better than the CT attenuation for predicting positive bone biopsies (*P* = 0.021). ADC performed better than the CT attenuation in this group, but this was not statistically significant (*P* = 0.084). There was no significant predictive difference between ADC and SIR (*P* = 0.407) in patients that never received any treatment.

Figure 1 shows a 75‐year‐old man with history of prostate cancer and a Tc^99^ MDP bone scan showing uptake in the right and left posterior iliac spine. A contemporary contrast enhanced CT (100kV, 315 mA) shows diffuse osteoblastic metastases involving the right greater than left posterior iliac spine, so the right posterior iliac spine was targeted for biopsy. Biopsy of the right posterior iliac spine showed predominantly acellular bone cores and no overt evidence of carcinoma. CSI‐MRI showed evidence of marrow replacement in the lesion in the left posterior iliac spine, but essentially normal marrow SIR in the lesion in the right posterior iliac spine. Subsequent DWI‐MRI of the pelvis showed no restricted diffusion in the right posterior iliac spine but restricted diffusion in a lesion in the left posterior iliac spine. Repeat biopsy, with biopsy of the lesion in the left posterior iliac spine confirmed metastatic prostate cancer.

## Discussion

The data show that the ADC and SIR values could be used to target lesions for biopsy in patients with bone lesions. A threshold ADC of 793 × 10^−6^ mm^2^/s had a sensitivity of 75.8%, and a threshold SIR of 0.949 had a sensitivity of 77.8% to detect malignancy. The data also show that the CT attenuation was significantly less predictive of malignancy than either the SIR or ADC of a lesion. We believe that quantitative MRI imaging (CSI‐MRI and DWI‐MRI) are valuable tools that can be utilised in planning bone biopsies, especially when a prior biopsy was negative.

Diffusion‐weighted imaging evaluates the random motion of water molecules in tissues, and the major factors influencing water diffusion include tissue cellularity and the presence of intact cell membranes.^15^, ^16^, ^17^ This suggests that the ADC maps can be used to identify the most cellular area with intact cell membranes to target for biopsy. Biopsy cellularity is crucial for histopathological and immunohistochemical studies. An acellular or paucicellular biopsy sample is likely challenging for the pathologist to evaluate and interpret. The cell morphology, cell nuclei, number of mitotic figures, extracellular matrix and degree of necrosis are important histological features used by pathologists for diagnosis, and several of these features are absent in acellular or paucicellular biopsy samples. CSI‐MRI detects marrow fat replacement, including by tumour cells.^13^ Therefore, the results suggest that lesions that show evidence of marrow fat replacement are highly likely to be malignant. The accuracy of chemical‐shift imaging decreases after treatment, likely because of healing/reparative response with reconstitution of normal marrow fat;^13^ therefore, the SIR can identify lesions that are untreated. Because there was no significant correlation between the SIR and ADC values, marrow fat replacement appears different from tumour cellularity, and suggests that the SIR and ADC are detecting different features of the bone lesion. Tumour cellularity as measured by the ADC maps, may be useful for bone biopsy planning. While ADC maps have the potential to be useful, CSI‐MRI is also an invaluable tool that can be used to select bone lesions for biopsy. This analysis shows chemical‐shift imaging not statistically significantly different from ADC maps for predicting malignancy from bone biopsies.

A prior study noted that the major factor associated with non‐diagnostic biopsies was the presence of lesional sclerosis, as seen with osteoblastic metastases.^10^ The CT appearance of an osteoblastic lesion is often not sufficient to determine whether a lesion was treated, partially treated or untreated, and for several cancers (breast, prostate, lymphoma and lung cancer), treatment results in increased sclerosis.^19^ Biopsies of treated/partially treated osteoblastic metastases may have contributed to the finding of lesional sclerosis being associated with non‐diagnostic biopsies in the literature. Although other factors related to the biopsy including tissue crush artefact, and factors related to the histological processing including decalcification may have also contributed to these findings. Lytic lesions (lesions with lower CT attenuation) were more likely to yield positive bone biopsies, but the AUC of the CT attenuation was marginally better than that expected by chance. A prior paper showed that a threshold mean CT attenuation of 885 HU had an accuracy of 91.7% and 81.7% to differentiate enostoses from untreated and treated osteoblastic metastases, respectively.^12^ This analysis was restricted to only osteoblastic lesions, whereas our analysis considered all bone lesions that were suspicious enough to be biopsied. Our study evaluated whether the CT attenuation of a bone lesion was predictive of whether it was malignant or not, which was a slightly different question.

We suspect that a combined metric utilising ADC, and SIR values may be more informative for predicting malignancy; however, this study was not powered to detect whether this approach would be superior to the ADC or SIR values alone. In addition, inclusion of a T2‐based metric may assist in predicting whether a bone biopsy shows malignancy, however, further research is required to assess this.

This study has a few limitations. First, the study sample size was limited. A variable number of biopsy cores were obtained at the discretion of the performing radiologist, which in conjunction with technical factors, may have affected the biopsy yield for each patient. There were slight differences in the diagnoses between patients with positive biopsies and patients with negative biopsies. Breast, bladder and lung cancers were more likely to yield positive biopsies than prostate cancer and lymphoma. Patients without a pre‐biopsy MRI study with diffusion‐weighted imaging and chemical‐shift imaging sequences were excluded. This may have biased the sample towards patients with cancers that are typically followed with MRI surveillance imaging rather than CT.

In conclusion, because DWI‐MRI and CSI‐MRI detect tumour cellularity and bone marrow fat replacement, respectively, both of these techniques can be used to help predict malignancy in bone biopsies.

## Funding Information

RS was supported by R21 NIH/NIMH MH093415.

## Conflict of interests

All authors have completed the ICMJE uniform disclosure form at www.icmje.org/coi_disclosure.pdf and declare: no financial relationships with any organisations that might have an interest in the submitted work in the previous three years; no other relationships or activities that could appear to have influenced the submitted work.
